# Metagenomic insights into particles and their associated microbiota in a coastal margin ecosystem

**DOI:** 10.3389/fmicb.2014.00466

**Published:** 2014-09-05

**Authors:** Holly M. Simon, Maria W. Smith, Lydie Herfort

**Affiliations:** Center for Coastal Margin Observation and Prediction, Institute of Environmental Health, Oregon Health and Science UniversityPortland, OR, USA

**Keywords:** metagenomics, particle-attached, free-living, microbial communities, estuary, coastal margin

## Abstract

Our previously published research was one of the pioneering studies on the use of metagenomics to directly compare taxonomic and metabolic properties of aquatic microorganisms from different filter size-fractions. We compared size-fractionated water samples representing free-living and particle-attached communities from four diverse habitats in the Columbia River coastal margin, analyzing 12 metagenomes consisting of >5 million sequence reads (>1.6 Gbp). With predicted peptide and rRNA data we evaluated eukaryotic, bacterial and archaeal populations across size fractions and related their properties to attached and free-living lifestyles, and their potential roles in carbon and nutrient cycling. In this focused review, we expand our discussion on the use of high-throughput sequence data to relate microbial community structure and function to the origin, fate and transport of particulate organic matter (POM) in coastal margins. We additionally discuss the potential impact of the priming effect on organic matter cycling at the land-ocean interface, and build a case for the importance, in particle-rich estuaries and coastal margin waters, of microbial activities in low-oxygen microzones within particle interiors.

## Introduction

**Particulate organic matter** (POM) is one of the most important components of total suspended matter in aquatic ecosystems. The origin, transformation and ultimate fate of POM have long been and continue to be topics of intensive scientific investigation (Simon et al., 2002). Initially the living biomass of primary producers, POM in marine, estuarine, lacustrine, and riverine waters is transformed into detritus and decomposed through dense colonization and activities of heterotrophic microorganisms (including bacteria, protists, and metazoans) as part of the microbial loop (Zimmermann-Timm, 2002; Crump et al., 2004). Plumes of dissolved organic matter (DOM) can also greatly extend the intense decomposition processes beyond the volume of the particles themselves (Kiorboe and Jackson, 2001). Because these detrital particles are composites of smaller primary components they are referred to as organic aggregates (Simon et al., 2002), and their characteristics are largely determined by the physical and biological properties of the environment (Hedges and Keil, 1999).

KEY CONCEPT 1. Particulate organic matter Organic aggregates vary in size (the two size classes discussed here include macroaggregates, which are >500 μm, and microaggregates, <500 μm) and composition [including living, senescent and dead biomass, various organic compounds, and gels (e.g., transparent exopolymer particles), as well as inorganic components] across ecosystems and environmental conditions. They are enriched in organic and inorganic nutrients when compared to the surrounding water, and have been identified as “hotspots” of microbial decomposition of organic matter.

Marine carbon fluxes are dependent upon the dynamics of DOM, thus, identifying the mechanisms regulating fluxes of POM to DOM is critical to understanding global carbon cycles (Simon et al., 2002). POM and DOM, respectively, are defined operationally as the material that is either retained, or passes through, a filter with a pore size of 0.22 μm (or, sometimes, 0.45 or 0.7 μm) (Simon et al., 2002; Jiao et al., 2010). POM is universally important in different aquatic environments for organic matter remineralization and elemental cycling, even though specific particle traits in these ecosystems vary. For example, marine and lake particles (termed “snow”) are large composites of organic-rich detrital matter, while estuarine particles tend to be smaller and contain significant amounts of inorganic material: silt, clay and sand with low organic matter content (Sherwood et al., 1984). Thus, rivers discharge an abundance of terrestrial organic matter to estuaries and the coastal ocean that contains a heterogeneous mix of vascular plant detritus, soil, and older fossil carbon eroded from rocks (Prahl et al., 1997; Turner and Millward, 2002). Nevertheless, labile, organic-rich particles from live and decaying algal blooms are also transported into the estuary with freshwater influx or tidally advected coastal waters, providing biomass for estuarine remineralization (Small et al., 1990; Roegner et al., 2011).

Therefore, both labile and more recalcitrant forms of organic matter are imported into estuaries with water from end members. In light of this fact, the “**priming effect**,” and recent discussions that stress its absence in the literature on aquatic ecosystems (Guenet et al., 2010; Bianchi, 2011), are relevant. The priming effect occurs when the addition of labile compounds leads to increased degradation of more recalcitrant forms of organic matter. Studied for many decades in soil, the phenomenon has been almost completely overlooked in aquatic systems. Organic macroaggregates with co-occurring mixtures of labile and recalcitrant terrestrial-derived organic matter, and particle-attached microorganisms, are proposed to be hotspots of the priming effect (Guenet et al., 2010). If this prediction turns out to be accurate, carbon budgets for particle-rich environments, such as those at land-ocean margins, may be impacted significantly. Furthermore, increasing levels of atmospheric CO_2_ appear to lead to enhanced priming effects in terrestrial ecosystems from elevated plant production and release of labile root exudates into the soil (Qiao et al., 2014). So, too, might the priming effect be enhanced in aquatic systems if, for example, higher CO_2_ levels lead to enhanced primary production (Riebesell et al., 2007). Thus, the priming effect has potentially global implications for turnover and release of carbon to the atmosphere.

KEY CONCEPT 2. Priming effect The “priming effect” occurs with the exogenous addition of labile compounds, which enhance degradative processes and result in the release of carbon and nitrogen from otherwise less reactive compounds. It may be widespread in ecosystems, such as estuaries, where additions of allochthonous organic carbon can dramatically alter microbial community composition and activities.

Although the relevant experiments are just beginning to be done in marine ecosystems (Guenet et al., 2010; Bianchi, 2011), a study by Guenet et al. (2014) demonstrated the priming effect using several different sources of soil organic matter in microcosm experiments. The effect was observed to be generally higher in aquatic vs. terrestrial, and in eutrophic vs. oligo-mesotrophic conditions. The priming effect may in fact account, in part, for the low detectable terrestrial organic matter signature in the ocean (Bianchi, 2011). However, it is also important to note that the current paradigm is shifting away from the idea that terrestrial organic matter is chemically recalcitrant to degradation because it is dominated by lignified (aromatic) plant material or nitrogen-poor, old and complex soil organic matter. Instead, growing importance is being placed on environmental controls of both biotic and abiotic decomposition rates for terrestrially derived organic matter in aquatic ecosystems (Hedges and Keil, 1999; Bianchi, 2011; Marin-Spiotta et al., 2014).

## Origin and fate of particulate matter in estuaries

The fate of particulate matter in estuaries is determined by circulation dynamics, water residence times, and sedimentation rates. As an example of a particle-rich ecosystem, the river-dominated Columbia River estuary has multiple scales of variability controlled by tides and the two dominant end members (river and ocean; Simenstad et al., 1990). Annual and inter-annual variability in forcing and the availability of nutrients affect biological processes and, in turn, the formation and composition of particles (Zimmermann-Timm, 2002; Chawla et al., 2008). Successive cycles of particle deposition and re-suspension lead to high turbidity and continuous chemical exchanges between the aqueous phase, suspended phase, and bed (Turner and Millward, 2002). The bulk of suspended particulate matter is mainly terrestrial in origin (Prahl et al., 1997), and overall, the Columbia River is estimated to supply >10 million tons of sediment per year to the estuary and coastal ocean (Sherwood et al., 1984). In addition to abiotic sediments and vascular plant detritus, the particle loads include expired freshwater phytoplankton that develop seasonally in the river starting from March (Small et al., 1990). Under late summer conditions with relatively low river discharge, large tidally-driven salinity intrusion also facilitates sediment flux into the estuary from adjacent continental shelf regions (Sherwood et al., 1984). During nearshore seasonal **upwelling** events, this salinity intrusion often carries coastal phytoplankton blooms, thereby supplying ocean-derived POM to the lower estuary (Kudela et al., 2005; Roegner et al., 2011; Herfort et al., 2011a). Extensive beds of large subtidal kelps (the brown algae Phaeophyta) that are a typical feature of the temperate Pacific Northwest coastline (Schapira et al., 2012) also supply POM along the coastal margin.

KEY CONCEPT 3. Upwelling Summer upwelling is observed near shore of the Eastern North Pacific Ocean as prevailing winds push the surface water away from the coastline. Water moving offshore is replaced by upwelled cold, high-salinity, and nutrient-rich water from depths of 150–300 m. The flux of nutrients to the upper euphotic zone enhances production and standing stocks of species throughout the food web.

In addition to allochthonous sources, autochthonous particles in the Columbia River estuary originate from estuarine blooms of protists—*Mesodinium* spp. in summer (Herfort et al., 2011b,c) and *Katablepharis* spp. in spring (Kahn et al., in press) Fine sediment particles from productive lateral bays are also found periodically in the main channels (Simenstad et al., 1984), and may be re-suspended and retained in the lower estuary due to development of large and often well-defined, but transient, **estuarine turbidity maxima** (ETM) (Prahl et al., 1997; Small and Prahl, 2004). The dynamics of these transient ETM events depend upon sediment supply, tidal mixing and estuarine stratification. ETM concentrate both mineral and organic particles and extend their residence time in an otherwise fast-flushing estuary with low water retention time (Crump et al., 1998; Small and Prahl, 2004). This increase in retention time is thought to promote development of highly active particle-associated microbial assemblages (Crump et al., 1999, 2004). The elevated bacterial production rates observed in ETM particle-attached fractions suggest that they serve as hotspots for degradation of POM and uptake of DOM (Crump et al., 1998). Additional support for this hypothesis was provided by our metagenome data (details below), which, by analysis with homology and Hidden Markov models methods, showed enrichment in the larger-size ETM fractions (relative to the smaller-size fraction) of bacterial genes involved in decomposition of phytoplankton and assimilation of diatom exopolysaccharides, as well as those involved in utilization of dissolved organic carbon (Smith et al., 2013). This type of study emphasizes the utility of environmental genomics for investigating the roles of particle-attached vs. free-living microorganisms in organic matter cycling.

KEY CONCEPT 4. Estuarine turbidity maxima Estuarine turbidity maxima (ETM) are transient events created by the interaction between river flow and tidal forcing that suspend and trap sediments and other particles. In the Columbia River estuary, ETM extend the residence time of particles beyond water residence times (typically one to a few days), facilitating development of estuarine-specific, particle-attached bacterial populations.

## Molecular analyses of particle-attached communities

Molecular characterization of particle-associated microbiota may reveal specific details about the mechanisms regulating fluxes of POM to DOM, which ultimately influence rates of carbon export and storage (Jiao et al., 2010). For example, diatom-bacterial interactions have been implicated in the control of phytoplankton growth dynamics, aggregation, and sinking during blooms (Grossart et al., 2006). The standard approach for collection and size-fractionation of microorganisms in water column samples is to pass them through a series of filters with decreasing pore sizes. This method was implemented during the Global Ocean Sampling Expedition (Rusch et al., 2007), with large water volumes (>200 L per sample), subsequent filtration, and collection of three size fractions, 0.1–0.8, 0.8–3, and 3–200 μm. After filtration, a number of techniques can be used to query microbial communities. These include: (i) next-generation sequencing techniques for whole DNA and/or RNA samples followed by taxonomic and metabolic profiling (**metagenomics** or metatranscriptomics, respectively) (Allen et al., 2012); (ii) analysis of 16S rRNA gene diversity (Bizic-Ionescu et al., 2014; D'Ambrosio et al., 2014); and (iii) fluorescence *in situ* hybridization and microscopy (Simon et al., 2002 and references within). Recent advances in single-cell genomics (Stepanauskas, 2012; Rinke et al., 2013) additionally hold exceptional promise for understanding the functional properties of particle-attached taxa.

KEY CONCEPT 5. Metagenomics Metagenome-scale analysis involves high-throughput sequencing of genetic material (DNA) isolated from environmental samples. This approach allows for relatively unbiased sampling of the genomes from a mixed microbial assemblage.

With the serial filtration technique, microbial cells appear mainly to fractionate according to their size. However, cross-contamination does occur, presumably from the cell-free DNA of dead and decaying cells. For example, DNA from eukaryotic chloroplasts was observed in estuarine samples collected with or without pre-filtering through a 1-micron screen, even though the host organisms were too large to pass through the screen (Crump et al., 2004). Furthermore, flocculation and aggregation of particles may result in cross-contamination with material from a different size class (Simon et al., 2002), and particle aggregation due to filter clogging can also lead to retention of small particles on larger-pore size filters. Another factor influencing the utility of this approach is our ability to distinguish particle-associated from larger-sized, free-living microorganisms that are captured in the particulate fractions. Even with these caveats, however, the method is providing insights into selective forces shaping aquatic microbial communities, as discussed in more detail below.

The first studies of particle-attached microbiota in the Columbia River estuary were carried out by Crump et al. (1998, 1999), who used bacterial production (calculated from rates of ^3^H-thymidine incorporation) and 16S rRNA clone diversity to compare free-living and particle-attached bacteria. We recently followed up on this research with a comparative metagenomic analysis of summertime samples collected from: (1) an ETM; (2) a chlorophyll maximum in the river plume; (3) an upwelling-associated shelf hypoxic zone; and (4) the deep ocean bottom (Smith et al., 2013). There have been only a few metagenome-scale studies thus far comparing particle-attached and free-living microbial communities in waters from: (i) the California Current and Southern California Bight (Allen et al., 2012); (ii) the Columbia River coastal margin (Smith et al., 2013); and (iii) an oxygen minimum zone in the Eastern Tropical South Pacific upwelling zone off Chile and Peru (Ganesh et al., 2014). In general, these studies revealed taxonomic and functional distinctions between filter size classes. Large-size fractions, representing particle-associated communities, contained a higher proportion of eukaryotic, viral and phylogenetically unclassified sequences (many putatively involved in DNA mobilization and signaling-related pathways), as well as a generally more diverse and complex gene repertoire relative to smaller-size fractions (Allen et al., 2013). Bacterial genomes were also generally larger, and transporters more abundant in larger-size fractions, indicating more nutrient-replete conditions for particle-associated compared to free-living bacteria. These observations suggest that oligotrophic and copiotrophic microorganisms are unequally distributed in the marine environment, being enriched in free-living, and particle-associated communities, respectively (Allen et al., 2013). Furthermore, larger-size fractions were overrepresented in genes mediating surface colonization and cell–cell interactions, which are presumably more important for growth and survival on particles (Ganesh et al., 2014). Some additional highlights from our work in the Columbia River coastal margin are described in the sections that follow.

## Differences in community composition and metabolic properties of particle-associated vs. free-living microorganisms

In many coastal systems, including coastal bays and harbors of the United States (DeLong et al., 1993; Noble et al., 1997), the Mediterranean Sea (Ghiglione et al., 2007), the Arctic (Garneau et al., 2008), and Hong Kong (Zhang et al., 2007), as well as estuaries (Crump et al., 1999; Karrasch et al., 2003), the structure of particle-attached bacterioplankton communities was found to differ significantly from associated free-living populations. On occasion, however, greater similarity was found between different size fractions (Hollibaugh et al., 2000; Moeseneder et al., 2001). Particle-attached bacteria are often observed to be larger than free-living bacteria, likely due to greater substrate accessibility compared to the surrounding waters (Alldredge et al., 1986; Simon et al., 2002). Many reports also document higher activity on a per-cell basis for particle-attached compared to free-living bacteria (Fandino et al., 2001; Grossart et al., 2003, 2007; Schapira et al., 2012). Others have, however, noted higher activities in free-living populations (Alldredge et al., 1986; Martinez et al., 1996; Schapira et al., 2012), thus associated environmental parameters and particle properties are likely to be important determinant factors.

The abundance and activities of particle-attached bacteria are of particular significance in the turbid waters of coastal margins (e.g., Crump et al., 1998). Our metagenome analysis of four coastal margin habitats showed differences between the size fractions representing particle-attached and free-living communities (Smith et al., 2013). For archaea, the most striking differences were observed in the putatively ammonia-oxidizing Thaumarchaeota populations, whose sequences were abundant in both hypoxic and deep ocean bottom water samples (>22,000 and >66,000 peptide sequence hits, respectively, up to 19% of all predicted prokaryotic peptides). These organisms appeared to be enriched in the two smaller-size fractions in the hypoxic zone, while they were most abundant in both the largest- and smallest-size fractions from deep water. Protein recruitment to the genome of “*Candidatus Nitrosopumilus maritimus*” SCM1 indicated 63 and 55% average sequence similarity (*p* < 0.001) for the hypoxic and deep water samples, respectively, and approximately 50% higher diversity in the deep water populations (Smith et al., 2013). In another study at depth (670 m) in the subtropical North Pacific gyre, Ingalls et al. (2006) used the natural distribution of radiocarbon in archaeal membrane lipids *in situ*, and an isotopic mass balance model to estimate the percentage of chemoautotrophic (83%) vs. chemoheterotrophic (17%) archaeal metabolism. Their data, however, were unable to distinguish separate populations of autotrophic and heterotrophic archaea from uniformly mixotrophic populations. Given the relatively high genomic diversity in our deep water populations, and corresponding enrichments in both particle-attached and free-living fractions, our results may suggest that separate populations with different metabolic capabilities exist in the deep ocean.

In our euphotic zone metagenomes, the four most abundant bacterial groups identified in family (≥60% identity) and genus (≥90% identity) level sequence annotations were similar to aerobic heterotrophs and photoheterotrophs belonging to the: (i) Flavobacteriaceae (Bacteroidetes/Flavobacteria); (ii) marine roseobacters, including Rhodobacterales spp. of the Rhodobacteraceae family (Alphaproteobacteria); (iii) SAR11 clade (Alphaproteobacteria); and (iv) marine Gammaproteobacteria belonging to the OM60/NOR5 and SAR92 clades (Smith et al., 2013). Size-fractionation of the photoheterotrophic taxa from these groups was generally consistent with results from other studies (Finkel et al., 2013), with putative proteorhodopsin (PR)-containing organisms enriched in free-living fractions and aerobic anoxygenic phototrophs (AAP) more abundant in particle-attached fractions. There were exceptions, however. For example, PR-containing representatives of SAR92 and *Dokdonia* were enriched in particle-attached fractions, and some Flavobacteria representatives were equally abundant in both free-living and particle-attached fractions (discussed further below).

Analysis of functional peptide categories by Clusters of Orthologous Genes (COG) annotations using the D-rank approach (Markowitz et al., 2008) to calculate normalized abundances across metagenomes indicated that proteins involved in utilization of dissolved organic carbon in coastal ecosystems were consistently underrepresented in the smaller-size fractions (Smith et al., 2013). These results included enzymes involved in amino acid, nucleotide and coenzyme transport (Poretsky et al., 2010; Rinta-Kanto et al., 2012). Also generally underrepresented in free-living (and overrepresented in particle-attached) fractions were functional gene groups that have been linked to decomposition of phytoplankton and assimilation of diatom exopolysaccharides (Smith et al., 2013), including TonB-dependent transporters and the carbohydrate-active enzymes α-mannosidase, α-L-fucosidase, and L-fucose permease (Teeling et al., 2012). Use of a method providing a more definitive link between phylogeny and function, such as single-cell genomics (Stepanauskas, 2012; Rinke et al., 2013), could have a transformative impact on our understanding of activities and interactions among members of particle-attached microbial communities.

## Large-size fractions contain molecular signatures of particle origin

Our study showed that the large-size fractions (3–200 μm) across samples contained up to 40% of sequences from multicellular eukaryotic organisms (Smith et al., 2013). These data were particularly useful for evaluation of particle origin—both the ETM and deep ocean bottom samples contained relatively abundant sequences representing the phylum Animalia—while sequences of marine diatoms were abundant in all three euphotic zone samples. This finding indicated that, in summer, these organisms were constituents of POM not only in the coastal ocean, but also in the estuary. These results were also similar to those of Herfort et al. (2011a), who analyzed pre-, peak, and post-ETM water using traditional (microscopy and biochemistry) and molecular (18S rRNA gene sequencing) methods to assess the origin of estuarine POM during the summer. Most of POM was refractory, and a large proportion was chlorophyll *a*-poor particulate organic carbon of mostly freshwater origin. Nevertheless, the labile POM fraction characterized by 18S rRNA gene sequencing revealed both allochtonous (marine and freshwater diatoms) and autochtonous (estuarine blooms of *Mesodinium major* and *Katablepharis* sp.) sources (Herfort et al., 2011a; Kahn et al., in press). Clearly molecular analysis of estuarine microeukaryotic composition can enhance our understanding of POM sources, particularly of the labile fraction, which in the study by Herfort et al. (2011a) was masked by the refractory signal when analyzed using a traditional biogeochemical approach.

Our metagenome data indicated that organic matter in the ETM was dominated by riverine and marine phytoplankton, with vascular plant debris comprising only a minor fraction (Smith et al., 2013). These results are similar to those reported by Prahl et al. (1997) from analysis of lignin phenol content. The vascular plant community composition, comprising 7–12% of all annotated eukaryotic peptide reads, was remarkably similar across all sampled habitats, including the deep ocean bottom sample collected away from the coast at 1500 m depth (Smith et al., 2013). The relatively high ratio of particulate organic carbon to nitrogen in that sample suggested that significant DNA degradation had taken place (Herfort et al., 2011a) in these deep waters. This result led us to speculate that the terrestrial plant DNA did not originate from vascular plant detritus, but instead from wind and riverine transport of lightweight, hydrolysis-resistant pollen and spores common to Pacific Northwest coastal plant communities (Heusser and Balsam, 1977). High sequence identity to reference genomes in anemophilous families (such as Pinaceae, Poaceae, and Brassicaceae) and Bryopsida (mosses) supported this idea (Smith et al., 2013). Our data were therefore consistent with the finding that only a small percentage of land-derived organic matter ends up in ocean water and sediments (Hedges et al., 1997).

As discussed above, the priming effect may be a factor contributing to the degradation of land-derived organic matter at coastal margins. Bianchi (2011) proposed that land-ocean margins with steep nutrient, light, and salinity gradients, and microbial communities that are highly adapted to changing physicochemical gradients, are likely to have high potential for the priming effect. Phytoplankton exudates from riverine and coastal blooms may contribute to priming in estuaries, which often receive relatively high inputs of recalcitrant terrestrial forms of carbon (Prahl et al., 1997; Turner and Millward, 2002). Furthermore, coastal upwelling zones, where “old” deepwater dissolved organic carbon comes in contact with productive surface waters may also have high priming effect potential (Bianchi, 2011).

## Does POM source determine diversity and activities of coastal margin microbial communities?

In particle-rich land margin ecosystems, shifting properties of POM may be determinants of compositional change in particle-associated bacteria, thereby influencing factors involved in the release and uptake of DOM. Becker et al. (2014) used high-performance liquid chromatography coupled to mass spectrometry (HPLC-MS) to detect and compare chemical features in DOM released by laboratory cultures of 8 marine phytoplankton species. *Prochlorococcus*, *Synechococcus*, and diatom taxa all produced complex patterns with unique and overlapping features to DOM from other strains. More closely-related taxa tended to produce more similar patterns of DOM. Given that the ability of microorganisms to process DOM is dependent upon a metabolic repertoire that varies across different taxa (Jiao et al., 2010), this work may have important implications for POM colonization and DOM uptake and utilization.

Fortunato et al. (2013) found that changes in water conditions and abundance of particulate organic carbon, particulate nitrogen and dissolved organic carbon were correlated with seasonal changes in abundant species serving as “indicators” for different regions of the Columbia River coastal margin. Indicator taxa in the river and estuary included members from the Actinobacteria, Flavobacteria, and Alpha- and Gammaproteobacteria classes. Although these authors did not particularly note the change in particle origin or character that occurs in the estuary with seasonal progression, i.e., from terrestrial sources to phytoplankton, and from riverine to coastal blooms, in an earlier study Crump et al. (2003) correlated seasonal changes in DOM source with shifts in bacterial community composition in an Arctic Lake.

**Figure 1:**
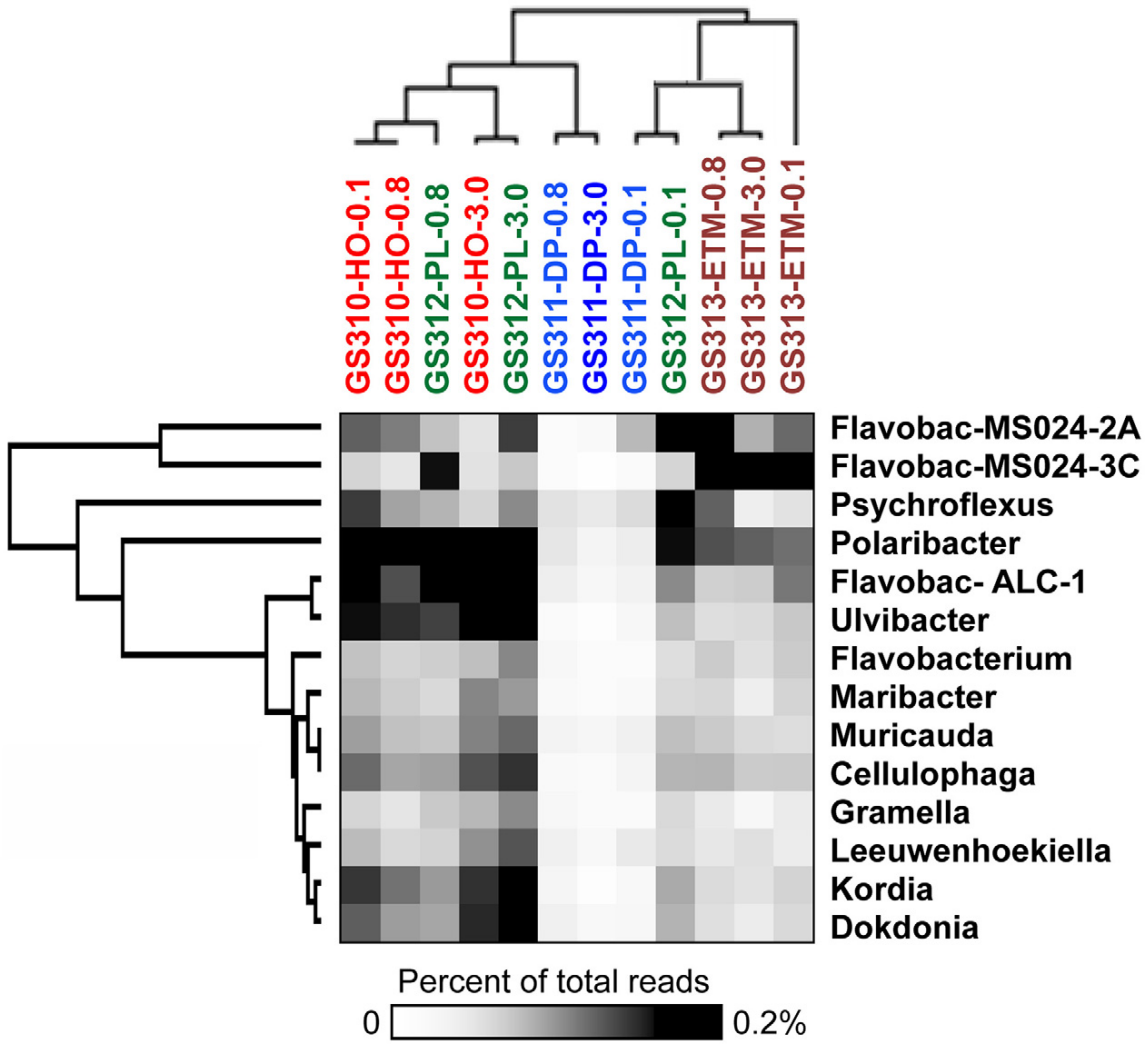
**2D Hierarchical clustering (average linkage) of genera from bacterial family Flavobacteriaceae (Y-axis) in metagenomes (X-axis)**. For each genus, abundance was calculated as the percentage of all corresponding hits with >90% identity to total annotated prokaryotic peptides in the corresponding metagenome. Abundance values are shaded from white to black on a scale indicating percentages from low to high. Sample names are composed of the GS (“global survey”) number of the JCVI sample data base and habitat: HO, shelf hypoxic water; DP, deep ocean bottom; PL, plume; ETM, estuarine turbidity maximum. The numbers above the sample names indicate size fractions: 0.1, 0.1–0.8 μm; 0.8, 0.8–3 μm; 3, 3.0–200 μm. Flavobac-ALC-1, Flavobacteriales bacterium ALC-1; Flavobac-MS024-2A, 3C, uncultured Flavobacteria.

Members of the Flavobacteria have been associated with phytoplankton particles and contain gene pathways involved in polysaccharide degradation (Kirchman, 2002; Karrasch et al., 2003; Teeling et al., 2012). These bacteria are particularly abundant during periods of high primary production (Williams et al., 2013). Moreover, in the coastal Arctic, algal blooms may stimulate community-level shifts to fewer free-living species and more particle-associated bacteria (Hodges et al., 2005). Closely-related diatom species or even the same algae at different growth stages may also harbor different microbial communities (Grossart, 2010), potentially providing insights into POM processing in marine and coastal waters. In our study (Smith et al., 2013), analysis by unsupervised 2D hierarchical clustering indicated that sequences representing many Flavobacteriaceae, including the genera *Ulvibacter*, *Dokdonia*, and *Cellulophaga*, and the unclassified *Flavobacterales* sp. ALC-1, were enriched in the large-size fractions of euphotic zone samples (Figure 1). *Ulvibacter* is associated with green algae and diatoms, and its abundance was dramatically enhanced during coastal diatom blooms in the North Sea (Teeling et al., 2012). The *Flavobacterales* sp. ALC-1 contains gene clusters putatively involved in degrading alginate, the main component of the kelp cell wall (Thomas et al., 2012). The larger-size fractions also tended to cluster together in this analysis, suggesting that the particle-attached representatives shared sequence similarities. In addition to genera enriched in specific fractions, a number of Flavobacteria representatives were abundant in both particle-attached and free-living fractions (Figure 1), e.g., *Polaribacter* and uncultured Flavobacteria MS024-3C). This suggests these organisms may be “generalists” (Kirchman, 2002; Grossart et al., 2007; Grossart, 2010), switching between free-living and particle-attached lifestyles depending upon environmental conditions (Kirchman, 2002; Teeling et al., 2012). The results in Figure 1 also indicate marine (e.g., *Kordia*, *Dokdonia*), estuarine (e.g., uncultured Flavobacteria MS024-3C) or broader (e.g., *Polaribacter*) distribution of particular Flavobacteria genera in the coastal margin. Note that Flavobacteria from this study were abundant only in euphotic zone samples, however, and almost completely absent from the deep ocean bottom water sample (Figure 1).

## Are low oxygen microzones in particles common features in turbid environments?

In an intriguing difference between the ETM and the other euphotic zone metagenomes, our data showed that a number of anaerobic and microaerophylic bacterial taxa, including Anaerolineaceae, Chlorobiaceae, bacterial sulfate reducers, and two Mn/Fe-reducing genera, *Pelobacter* and *Geobacter*, were greatly enriched in the ETM large-size fraction and all deep water metagenomes (Smith et al., 2013). These taxa were not abundant in either the corresponding ETM free-living metagenome, or in the plume or hypoxic water particulate fractions. The abundance of specific taxa was, in some cases, similar between the ETM and deep water metagenomes, and their overall abundance (up to ~4%) indicated that these organisms may have been actively growing. These results suggest the presence of low-oxygen microzones in suspended aggregates in the oxygenated water column. Additional results from analyzing whole water samples collected in the Columbia River estuary revealed: (i) identification of anaerobes, including anaerobic green sulfur bacteria of the phylum Chlorobi (Crump et al., 1999), and methanogenic archaea (Crump and Baross, 2000) by analysis of 16S rRNA gene composition; (ii) 1.5 to 2X higher average expression on microarrays, in estuarine relative to freshwater samples, of genes from various taxa utilizing dissimilatory nitrate reduction pathways (Smith et al., 2010); and (iii) enrichment of functional genes from dissimilatory sulfur and nitrogen reduction pathways in the ETM large-size fraction and deep water metagenomes (Smith et al., 2013). With respect to the latter, Ganesh et al. (2014) noted a similar trend in particle-associated metagenomes from an oxygen minimum zone.

In addition to the molecular genetic data described above, results of a study by Klinkhammer and McManus strongly suggested production of reduced manganese (Mn) in the Columbia River estuarine water column (Klinkhammer and McManus, 2001). A mid-salinity Mn maximum was found to correspond to the depth of the highest measured bacterial production in ETM (Crump et al., 1998) and occurred just below the zone of light penetration. The Mn maximum persisted at this depth throughout the estuary, and cut across density boundaries, suggesting *in situ* production that was estimated to account for 16% of the Mn entering the estuary. The authors suggested that particle-attached bacteria may produce these results through reduction of Mn oxides within the anoxic interiors of particles. Furthermore, advection from any source, including the lateral bays, could not explain the Mn anomaly, and its accumulation at mid-depth in the estuary suggested it was not due to significant inputs from groundwater (Klinkhammer and McManus, 2001).

Research by Alldredge and Cohen (1987) revealed the existence of O_2_ gradients around and within marine snow, and even anoxia within large fecal pellets upon incubation in the dark. The authors argued that bacterial respiration, which was at maximum at the particle surface, was sufficient to maintain microzones of O_2_ depletion, even within sinking particles with enhanced potential mass transfer of O_2_. Furthermore, Paerl and Prufert (1987), Paerl and Carlton (1988) measured enhanced nitrogenase activity and O_2_ gradients associated with aggregates, and suggested that N_2_ fixation in surface waters is largely dependent upon the availability of reduced microzones. Aggregation and nitrogenase activity were furthermore enhanced by additions of detrital matter (Paerl and Prufert, 1987; Paerl and Carlton, 1988). In their review, Riemann et al. (2010) summarized results from these and other studies supporting the idea that marine snow particles are ephemeral loci for N_2_ fixation by non-Cyanobacteria. Others, however, have also measured depleted O_2_ microzones in particles but contend that this phenomenon is unimportant in oxygenated water columns, where particles tend not to be limited at the aggregate-water interface by mass transfer and solute exchange processes (Ploug, 2001; Simon et al., 2002; Ploug et al., 2008). Anaerobic processes involving dissimilatory nitrate reduction have been measured in particles in the River Rhone plume and coastal waters of the northwestern Mediterranean Sea (Omnes et al., 1996; Michotey and Bonin, 1997), but the low rates detected are consistent with the idea that they may be ephemeral.

## Particulate low-oxygen microzones—transient but recurring phenomena in coastal margins?

**Figure 2:**
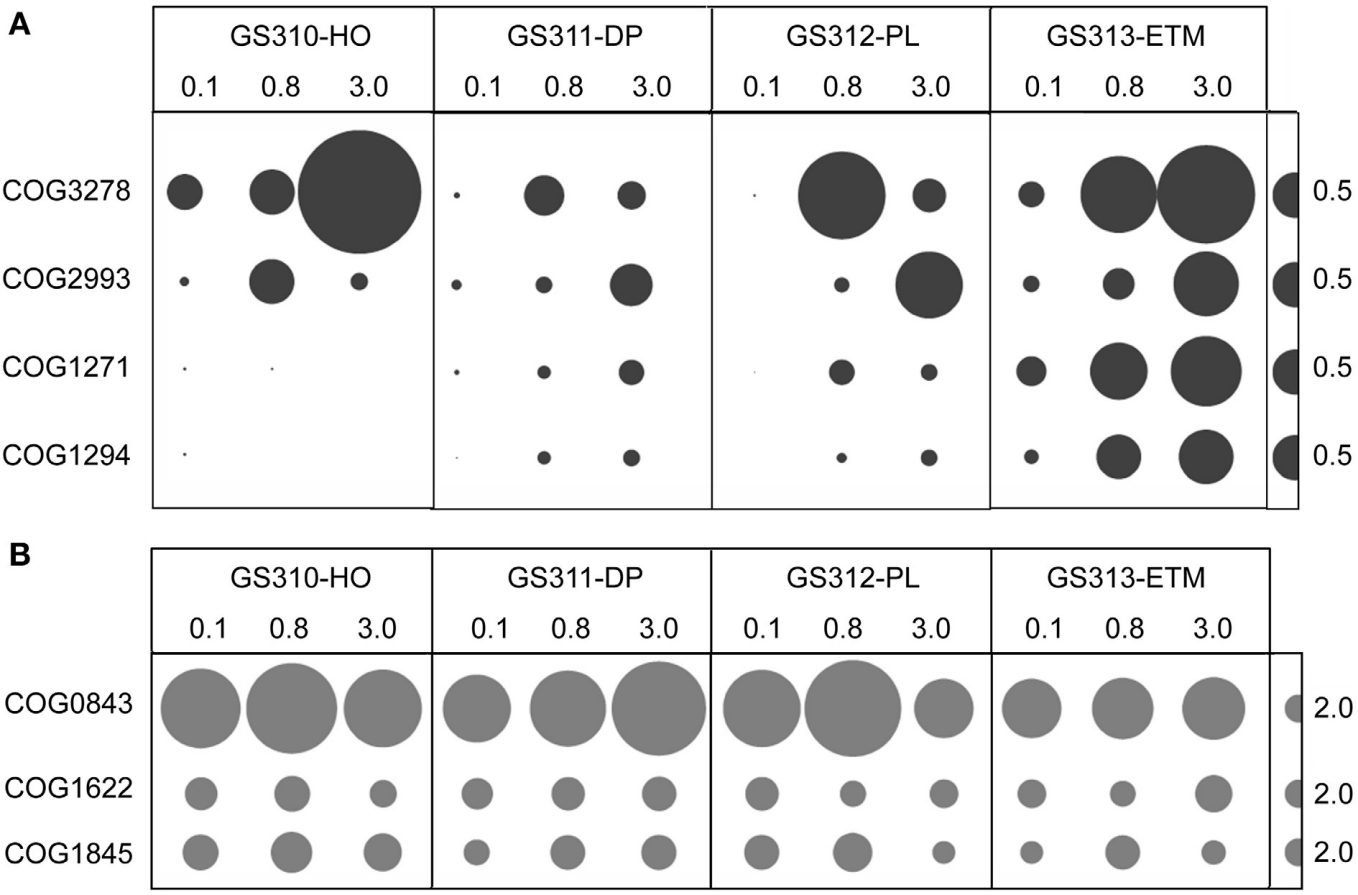
**(A)** High-affinity terminal oxidase gene categories: COG3278, Cbb3-type cytochrome oxidase, subunit 1; COG2993, Cbb3-type cytochrome oxidase, cytochrome c subunit; COG1271, Cytochrome bd-type quinol oxidase, subunit 1; COG1294, Cytochrome bd-type quinol oxidase, subunit 2. **(B)** “Housekeeping” heme/copper-type cytochrome/quinol oxidases: COG0843, subunit 1; COG1622, subunit 2; COG1845, subunit 3. Abundance for a functional gene category was calculated as the number of hits to a given category normalized by average bacterial genome equivalents in the corresponding metagenome. Abundance values are shown by bubble width for each size fraction in each sample. Half-bubbles to the right of each row correspond to 0.5 and 2 genes per average bacterial genome equivalent, in **(A,B)**, respectively. Sample names above the bubble plots are composed of the GS (“global survey”) number of the JCVI sample database and habitat: HO, hypoxic water; DP, deep ocean bottom; PL, plume; ETM, estuarine turbidity maximum. The numbers above the sample names indicate size fractions: 0.1, 0.1–0.8 μm; 0.8, 0.8–3 μm; 3, 3.0–200 μm.

Nevertheless, the data keep bringing us back to the question of whether low-oxygen microzones in particles are important to biogeochemical cycling at coastal margins. Could their existence influence the diversity of microorganism involved in, and the rates of, carbon, nitrogen and metal cycling? A recent report (Morris and Schmidt, 2013) highlights the presence of high-affinity terminal oxidase genes in sequenced bacterial genomes and shotgun metagenomes. The results indicate that bacteria with the potential to respire under microoxic conditions are more phylogenetically diverse and environmentally widespread than previously appreciated. Estuarine environments were, unfortunately, not included in this study. However, COG analysis of our own metagenome data indicated that the larger-size fractions of the ETM contained a similar abundance of these putative high-affinity terminal oxidase subunits compared to those found in soil, sediment, and mammalian gut habitats. The ETM sequences were also much higher in abundance than those identified in the marine environments examined (Figure 2, Morris and Schmidt, 2013).

Some of the data leading to predictions of O_2_ microzones in particles are potentially explained as residual carryover from re-suspended sediments present in the water column and trapped in the ETM. However, results from Mn and gene expression studies are more difficult to explain by that mechanism. As discussed earlier, researchers failed to find an outside source for the reduced Mn detected in the Columbia River estuarine water column (Klinkhammer and McManus, 2001). Also mentioned above, our study (Smith et al., 2010) examining gene expression by hybridization of cDNA on DNA microarrays (no amplification involved) suggested that anaerobic pathways were being expressed in the water column. Because mRNA is subject to intensive degradation and is relatively short-lived, these results are less likely to be explained by carryover from sediments. Although we do consider it likely that some transient carryover of sediment activities may be detected, we suggest that microaerobic metabolisms within suspended particulate matter may, in fact, impact carbon and nutrient cycling in the water column under certain circumstances. In particular, these conditions may occur during high rates of POM remineralization by bacteria, combined with intensive grazing by zooplankton species. Their combined activities may result in substantial localized drawdown of O_2_, limiting diffusion to the interior of particles. In support of this idea, the O_2_ consumption rate in rotifers increases upon feeding (Hirata and Yamasaki, 1987; Galkovskaya, 1995). Additionally, mesozooplankton respiration when grazing on phytoplankton near the base of the oxycline, in the oxygen minimum zone in the coastal upwelling zone off Chile, has been estimated in simulated field experiments to be sufficient under strongly stratified conditions to promote and maintain a persistent subsurface oxygen-deficient ecological barrier (BEDOX; Donoso and Escribano, 2014). The abundance of mesozooplankton has been correlated with suspended particulate matter and bacterial abundance in estuaries (Crump and Baross, 1996). Combined with elevated rates of bacterial production and respiration (Simon et al., 2002) and high particle loads at coastal margins, intensive grazing by micro- and mesozooplankton on bacteria and phytoplankton blooms (Crump et al., 2013) may facilitate the recurrent formation of low-oxygen microzones in particles. If so, over time this could cumulatively impact organic matter cycling in coastal margin ecosystems.

## Concluding remarks

In this focused review on particle-associated microbiota, we have attempted to relate DNA sequence analysis of community structure and function to the cycling of organic matter in land margin ecosystems. In many habitats, particle-associated microorganisms appear to be distinct from those in free-living fractions (DeLong et al., 1993; Crump et al., 1999; Simon et al., 2002; Eloe et al., 2011). Thus, analysis of microbial community composition may provide valuable insights into particle origin, transport, and fate. We additionally discussed the idea that allocthonous sources of POM may result in large priming effects in turbid estuarine and coastal margin waters, and presented evidence for the potential importance of low-oxygen microzones in particles to organic matter cycling. Both experimental, and modeling approaches will be useful in testing such ideas. Experimentally recreating the conditions that particles are subjected to in these dynamic environments would be challenging, but including grazers in experiments designed to measure O_2_ fluxes in sinking aggregates (such as in Ploug, 2001; Ploug et al., 2008) could provide evidence for low-oxygen microzone formation. For example, O_2_ diffusion to the interior of particles may be limited by localized drawdown from zooplankton grazing at particle exteriors. Low-oxygen, or even anoxic microzones may also be sustained for longer periods when zooplankton respiration rates are higher. Metatranscriptomic or reverse transcription PCR analysis of functional gene expression patterns in particle-attached bacteria during periods of intensive grazing may also be informative. Increased expression of metabolic genes involved in anaerobic pathways under these conditions would support our hypothesis. Finally, biogeochemical rate measurements, such as those carried out on particles in the River Rhone plume and coastal Mediterranean Sea (Omnes et al., 1996; Michotey and Bonin, 1997), could provide definitive evidence for anaerobic activities.

### Conflict of interest statement

The authors declare that the research was conducted in the absence of any commercial or financial relationships that could be construed as a potential conflict of interest.
